# Genome-wide analysis of aberrant methylation of enhancer DNA in human osteoarthritis

**DOI:** 10.1186/s12920-019-0646-9

**Published:** 2020-01-03

**Authors:** Xiaozong Lin, Li Li, Xiaojuan Liu, Jun Tian, Weizhuo Zheng, Jin Li, Limei Wang

**Affiliations:** 1 Department of Orthopedics, the Second Affiliated Hospital of Harbin Medical University, Harbin, 150081 China; 2 Department of Nuclear Medicine, the First Affiliated Hospital of Harbin Medical University, Harbin, 150001 China; 3 Department of Rehabilitation, the First Affiliated Hospital of Harbin Medical University, Harbin, 150001 China; 4 College of Bioinformatics Science and Technology, Harbin Medical University, Harbin, 150081 China; 5 College of Automation, Harbin Engineering University, Harbin, 150001 China

## Abstract

**Background:**

Osteoarthritis is a chronic musculoskeletal disease characterized by age-related gradual thinning and a high risk in females. Recent studies have shown that DNA methylation plays important roles in osteoarthritis. However, the genome-wide pattern of methylation in enhancers in osteoarthritis remains unclear.

**Methods:**

To explore the function of enhancers in osteoarthritis, we quantified CpG methylation in human enhancers based on a public dataset that included methylation profiles of 470,870 CpG probes in 108 samples from patients with hip and knee osteoarthritis and hip tissues from healthy individuals. Combining various bioinformatics analysis tools, we systematically analyzed aberrant DNA methylation of the enhancers throughout the genome in knee osteoarthritis and hip osteoarthritis.

**Results:**

We identified 16,816 differentially methylated CpGs, and nearly half (8111) of them were from enhancers, suggesting major DNA methylation changes in both types of osteoarthritis in the enhancer regions. A detailed analysis of hip osteoarthritis identified 2426 differentially methylated CpGs in enhancers between male and female patients, and 84.5% of them were hypomethylated in female patients and enriched in phenotypes related to hip osteoarthritis in females. Next, we explored the enhancer methylation dynamics among patients with knee osteoarthritis and identified 280 differentially methylated enhancer CpGs that were enriched in the human phenotypes and disease ontologies related to osteoarthritis. Finally, a comparison of enhancer methylation between knee osteoarthritis and hip osteoarthritis revealed organ source-dependent differences in enhancer methylation.

**Conclusion:**

Our findings indicate that aberrant methylation of enhancers is related to osteoarthritis phenotypes, and a comprehensive atlas of enhancer methylation is useful for further analysis of the epigenetic regulation of osteoarthritis and the development of clinical drugs for treatment of osteoarthritis.

## Background

Osteoarthritis (OA) is a chronic musculoskeletal disease that affects 250 million people worldwide [1]. OA is characterized by age-related gradual thinning. In addition to age, several other factors, including obesity, behavioral influences, and both nuclear and mitochondrial genetics, are related to this disease. This disease also shows sex differences in the prevalence, incidence, and severity, with females generally at a higher risk than males, which has been known for many years [2, 3]. Nevertheless, the molecular mechanism underlying the sex difference is still unknown. Molecular analysis revealed that the development of OA is related to gene mutations. For example, genome-wide association studies and analysis of quantitative trait loci have identified many candidate genes at loci associated with a risk of hip and knee OA [4, 5]. However, there are some clinical cases with no OA-related mutations suggesting the potential roles of epigenetic factors in OA development [6].

Epigenetics refers to stable heritable traits that cannot be explained by changes in DNA sequence. While the genetic code is the same for somatic cells, epigenetic changes have been found across individuals, tissues, and even cells from the same tissue. Epigenetic mechanisms include DNA methylation and many kinds of histone modifications. Among these mechanisms, DNA methylation is the best characterized epigenetic modification and involves the addition of methyl groups to cytosines, predominantly at the dinucleotide CpG. It has been reported that CpG methylation undergoes dynamic changes in development and throughout the lifespan of an individual and is also altered by environmental factors. DNA methylation has important roles in gene regulation, and its aberration has been observed in many kinds of human diseases, including cancers, since the 1990s [7].

Enhancers have important roles in gene regulation. The enhancer-promoter interaction can enhance the expression of downstream genes [8]. Most recent studies have revealed methylation dynamics in enhancer regions and their roles in regulating tissue-specific gene expression [9–11]. Liu et al. identified cell type-specific hypomethylation marks that were associated with cell type-specific superenhancers that drive the expression of genes associated with cell identify [11]. Abnormal methylation patterns in enhancers contribute to abnormal gene expression in multiple diseases, including many kinds of cancers [12].

Recently, some studies have revealed abnormal DNA methylation in OA, particularly methylation dynamics in gene promoters [13–17]. These studies provide a systemic view of the DNA methylation changes in gene promoters and their roles in OA development. For example, Moazedi-Fuerst et al. profiled methylation in 15 female OA patients using human promoter microarrays [13]. Fernández-Tajes et al. detected promoter methylation in 25 OA patients using Illumina Infinium HumanMethylation27 arrays in which the ~ 27,000 probes were designed for gene promoters [14]. Aref-Eshghi et al. enlarged the coverage of CpG sites by using an Illumina Infinium HumanMethylation450 BeadChip array, which includes newly added probes targeting enhancer regions other than those in HumanMethylation27; however, the sample size was relatively small (5 patients with hip OA, 6 patients with knee OA and 7 hip cartilage samples) [15]. Rushton et al. profiled DNA methylation in a large cohort (23 patients with hip OA, 73 patients with knee OA, and 21 control patients with healthy hips) by the HumanMethylation450 array and revealed genome-wide methylation changes in the OA patients [16]. Nevertheless, none of these studies focused on enhancer regions, and the genome-wide enhancer methylation patterns in OA remain unclear.

To this end, we quantified CpG methylation in human enhancers based on a public dataset that included methylation profiles of 470,870 CpG probes in 108 human samples from patients with hip and knee osteoarthritis and controls with healthy hips. We investigated the enhancer methylation landscape in OA patients and their roles in regulating OA development. Systemic analysis revealed the methylation dynamics in enhancers in two kinds of osteoarthritis, knee osteoarthritis and hip osteoarthritis. Our findings revealed major changes in DNA methylation in enhancers and their correlation with human phenotypes related to osteoarthritis. The comprehensive enhancer methylation atlas proposed in this study is useful for further analysis of the epigenetic regulation of osteoarthritis.

## Methods

### DNA methylation data

**Fig. 1:**
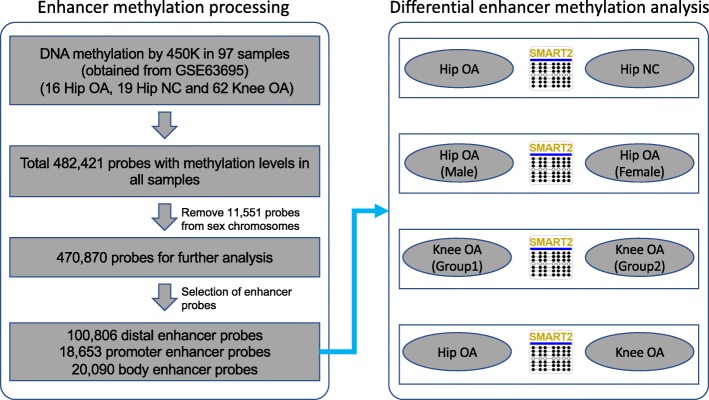
Schematic of the procedure for this study. The left panel shows the DNA methylation processing and enhancer probe selection. The right panel shows the differential methylation analysis between each pair based on SMART2

The DNA methylation data were downloaded from the GEO database under access ID GSE63695 [16]. In total, 97 samples of cartilage chondrocytes were obtained from three groups of patients with primary hip OA (*N* = 16) and primary knee OA (*N* = 62) and healthy controls (NC) without any OA disease in the hips (*N* = 19). All DNA methylation profiles were detected by an Illumina Infinium HumanMethylation450 BeadChip array (450 K). The annotation file of this array was downloaded from the GEO database under access ID GPL13534. DNA methylation values, described as “β values”, were calculated as M/(M + U), where M represents the fluorescent signal of the methylation probe and U represents the signal of the unmethylated probe. The β values range from 0 (no methylation) to 1 (full methylation). In total, there are 482,421 probes with methylation levels in all samples. To avoid the influence of X chromosome inactivation in the female sample, we removed 11,551 probes targeting the sex chromosomes, and 470,870 probes remained for further analysis (Fig. 1).

### Classification of the enhancer probes

We obtained the CpG probes localized in distal enhancer regions from the supplemental file of a previously published reference [18]. From the remaining CpG probes, we selected 100,806 distal enhancer probes that have methylation values in all samples (Fig. 1). Furthermore, we obtained 18,653 promoter enhancer probes shown to be localized near gene transcript start sites (TSSs), which were tagged TSS1500, TSS500, TSS200, 5’UTR and first exon in the 450 K annotation. As recently reported, the enhancers in the gene body also play important roles in gene regulation [19]; thus, we selected 20,090 enhancer probes that overlapped with gene body regions. Detailed information on these enhancers can be found in Additional file 3: Table S1.

### Identification of differentially methylated CpGs in enhancers

To identify the differentially methylated CpGs (eDMCs) in enhancers among the three main groups, including the hip OA, knee OA and NC groups, we used the recently developed tool SMART (Fig. 1), which was designed for the identification of differentially methylated sites or regions for bisulfite-based DNA methylation data [11]. The default parameters were used for eDMC calling. Furthermore, a two-way t test was used for pairwise comparison of methylation differences between two groups. An eDMC between two groups was identified if the *p* value was less than 0.05 and the absolute methylation difference was larger than 0.2. For the eDMCs between the two groups in patients with knee OA, those with *p* values less than 10^− 10^ were also identified as eDMCs, even with absolute methylation differences less than 0.2.

### Hierarchical cluster and function enrichment

All hierarchical clusters were determined out by Cluster 3.0, and the heatmaps were visualized by TreeView 3.0 [20]. All functional enrichment of eDMCs was carried out by GREAT, which is a tool for enrichment of annotations of genomic regions [21], using the default parameters.

### Statistical analysis

Statistical analysis was performed using R. Comparison between groups was performed using Student’s t test.

## Results

### The methylation patterns of enhancers in patients with OA and healthy controls

**Fig. 2:**
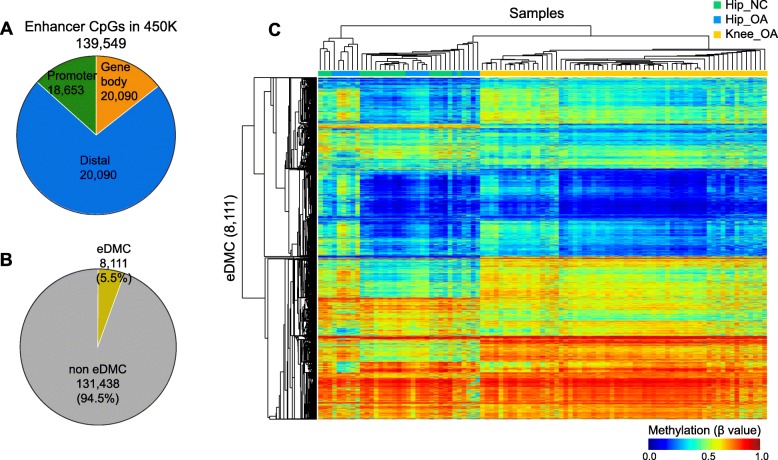
DNA methylation patterns of differentially methylated enhancer CpGs (eDMCs) in patients with hip OA, patients with knee OA and healthy controls. **a** The composition of enhancer CpGs in different genome contexts included in the 450 K BeadChip array. **b** Fraction of the eDMCs identified by SMART in the three groups. **c** Two-way hierarchical clustering and heatmap of the DNA methylation values of the eDMCs across all samples. DNA methylation (β value) is represented by a color from dark blue (unmethylated) to dark red (fully methylated)

To explore the methylation pattern in OA patients, we obtained the methylation values of 470,870 CpG probes detected in all samples from three groups, including patients with hip OA, patients with knee OA and healthy controls, by an Illumina Infinium HumanMethylation450 BeadChip array (450 K). In this array, 139,549 of these CpGs were from human enhancer regions. According to their locations relative to genes, these enhancers were classified into three groups: promoter enhancer around the transcript start site (TSS), gene body enhancer and distal enhancer distant from the gene TSS (Fig. 2a).

To analyze the enhancer methylation pattern, we used SMART to identify the differentially methylated CpG sites (DMCs) across three groups from all available CpGs in the human genome. In total, 3.6% (16,816) of the CpGs were identified as DMCs. Nearly half (8111) of the DMCs are from enhancers, which was significantly higher than expected (Chi-squared test *p* < 2.2e-16) (Fig. 2b). A high percentage of enhancer DMCs (eDMCs) suggested that the enhancer regions undergo more methylation than other genomic regions. To assess this notion intuitively, we carried out two-way hierarchical clustering to show the methylation patterns in the samples. As shown in Fig. 2c, the samples were classified into three clusters. We found mixed clustering among the hip OA samples and hip NC samples, which suggests the existence of hip OA subsets. In addition, we found two obvious subsets in the knee OA groups, although all knee OA samples were clustered into the same cluster. These results prompted us to explore the detailed methylation changes in each group.

### Gender-dependent methylation dynamics among the patients with hip OA

**Fig. 3:**
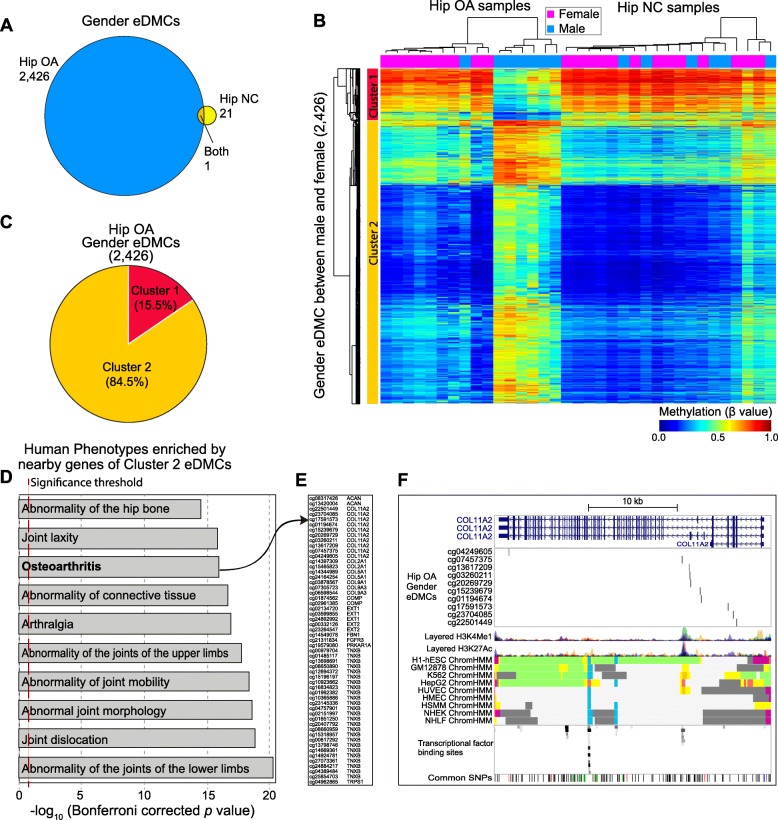
Gender-specific enhancer methylation related to osteoarthritis phenotypes in hip osteoarthritis. **a** The number of gender-specific eDMCs identified in the hip OA group and hip NC group. **b** Two-way hierarchical clustering of methylation in the gender-specific eDMCs across hip samples. Each row represents a gender-specific eDMC, and each column represents a sample colored by two colors: purple for females and blue for males. DNA methylation (β value) is represented by a color from dark blue (unmethylated) to dark red (fully methylated). **c** The fraction of the eDMCs in cluster 1 and cluster 2. **d** Human phenotypes enriched by genes near the eDMCs in cluster 2. The length of each bar represents the significance of the phenotype enriched by eDMCs in cluster 2. Detailed information can be found in Additional file 4: Table S2. **e** The eDMC-gene pair for the eDMCs enriched in the osteoarthritis phenotype. **f** The genome context of the eDMCs related to the gene COL11A2, which was made by the UCSC genome browser

There are sex differences in the prevalence, incidence, and severity of OA. The molecular mechanisms underlying these phenomena are still not clear. To this end, we compared the differences in enhancer methylation between the male and female patients in the hip OA group and identified 2426 eDMCs. As a control, we compared methylation between the male and female patients in the hip NC group and identified 21 eDMCs, including one common eDMC (Fig. 3a). In addition, we compared the differences in enhancer methylation between the male and female patients in the knee OA group, and no eDMCs were identified. These results suggested that gender-dependent differences in enhancer methylation only occurred in patients with hip OA. Two-way hierarchical clustering based on the methylation values of the gender-related eDMCs also showed gender-dependent clustering of the hip OA samples, while the hip NC samples did not cluster according to gender (Fig. 3b). Meanwhile, the gender-related eDMCs were classified into two clusters. Approximately 15.5% of the gender-related eDMCs showed hypomethylation in male patients, while 84.5% showed hypomethylation in female patients (Fig. 3c).

Enhancers can interact with promoters in the same topologically associating domain, which ranges in size from thousands to millions of DNA bases and enhances the expression of related genes [22]. Thus, the genes localized near the eDMCs can be used to speculate about the potential functions of the gender-related eDMCs. To this end, GREAT software [21] was used to explore the functions enriched by the gender-related eDMCs in two clusters. Notably, the gender-related eDMCs in cluster 2 were significantly enriched in some human phenotypes related to joints, including osteoarthritis, while those in cluster 1 were not (Fig. 3d and Additional file 4: Table S2). This finding suggested that the higher OA risk in females than males was related to the methylation changes in enhancers. The genes related to the human phenotype of osteoarthritis include well-known genes that have been shown to be related to osteoarthritis in previous studies (Fig. 3e). For example, Ritvaniemi et al. found that a mutation in *COL11A2* can result in early-onset osteoarthritis [23]. Here, we showed new evidence of gender-specific methylation of ten CpG sites from the enhancer regions of *COL11A2,* which may be related to gender-specific phenotypes. To confirm the enhancer status in these gender-specific eDMCs, we assessed the enhancer marks around these CpG sites. As shown in Fig. 3f, nine of these gender-specific eDMCs were enriched by the enhancer marks H3K4me1 and H3K27ac, transcription factor binding sites. In addition, this region shows enhancer activity in other disease cell lines, including an immortalized myelogenous leukemia line (K562), a liver cancer cell line (HepG2) and human umbilical vein endothelial cells (HUVECs). Integrating DNA methylation profiles in cancer cell lines obtained from the ENCODE project, we found that male OA patients showed similar methylation patterns to cancer cell lines (Additional file 1: Figure S1), suggesting distinct molecular mechanisms between male OA and fetal OA. These results revealed the gender-specific enhancer methylation related to osteoarthritis phenotypes in hip osteoarthritis for the first time.

### Two subtypes of patients with knee OA identified based on enhancer methylation

**Fig. 4:**
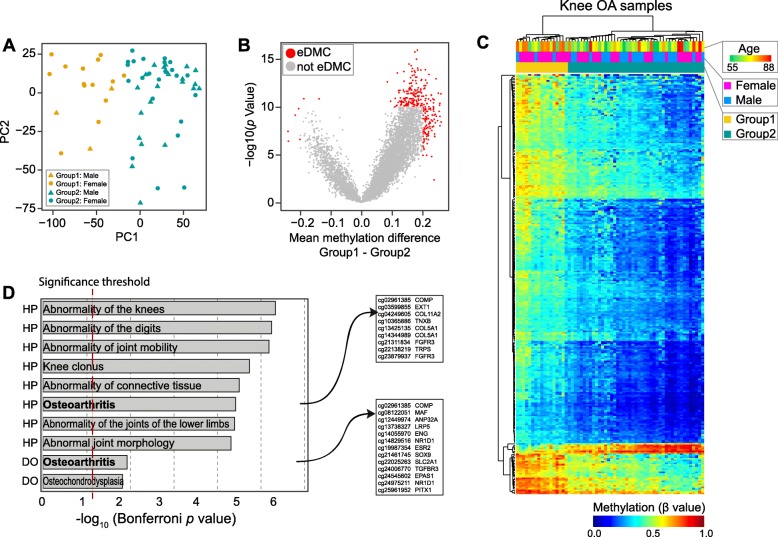
Subtypes of patients with knee OA showed differences in enhancer methylation related to osteoarthritis phenotypes. **a** Principal component analysis of patients with knee OA based on the methylation profiles of 8111 DMCs. First, two principal components (PC1 and PC2) were used to show the classification of the knee OA samples. **b** Volcano plot of the mean methylation differences between two knee groups. Red dots represent the eDMCs identified by t tests. **c** Two-way hierarchical clustering of methylation in group-specific eDMCs across the knee OA samples. Each row represents a group-specific eDMC, and each column represents a sample. For each sample, the age is represented by a gradual color from green (young) to red (old), the gender is indicated by purple for females and blue for males, and the group was colored by two colors that are the same as those in Figure **a**. DNA methylation (β value) is represented by a color from dark blue (unmethylated) to dark red (fully methylated). **d** Human phenotypes (HP) and disease ontologies (DO) enriched by nearby genes of the eDMCs. The length of each bar represents the significance of the phenotype enriched in the eDMCs. The right panel shows the eDMC-gene pair for the eDMCs enriched in osteoarthritis as a human phenotype and disease ontology

As shown in Fig. 2c, the knee OA patients were classified into two groups. To confirm this, we carried out principal component analysis on the patients with knee OA based on the methylation profiles of 8111 DMCs. We found that the OA patients can be classified into two groups by the first two principal components (Fig. 4a). To further analyze the DNA methylation pattern underlying this phenomenon, we identified the eDMCs between the two groups. In total, 280 CpG sites were identified as eDMCs showing methylation differences between the two groups. As shown in Fig. 4b, nearly 97.5% of these eDMCs showed higher methylation levels in Group 1. Two-way hierarchical clustering based on the CpG methylation profiles of 280 eDMCs revealed hypermethylation in Group 1 (Fig. 4c). We found that 82.4% of the patients in Group 1 were female, which was significantly higher than that (51.1%) in Group 2, suggesting gender bias of methylation in the patients with knee OA. We also found three male patients in Group 1. Further analysis of their ages revealed that these male patients were older than those in Group 2, although the results from the Mann-Whitney-Wilcoxon test were not significant due to insufficient samples in Group 1 (Additional file 2: Figure S2). To confirm the potential functions of these group eDMCs between the knee OA subtypes, we performed functional enrichment via GREAT. The results showed the enrichment of these eDMCs in the human phenotypes and disease ontologies related to osteoarthritis (Fig. 4d and Additional file 5: Table S3). For example, an eDMC (cg02961385) was localized near a gene, *COMP,* which was shown to be a novel diagnostic and prognostic biomarker for knee osteoarthritis [24].

### Differences in organ source-dependent enhancer methylation between the patients with hip OA and knee OA

**Fig. 5:**
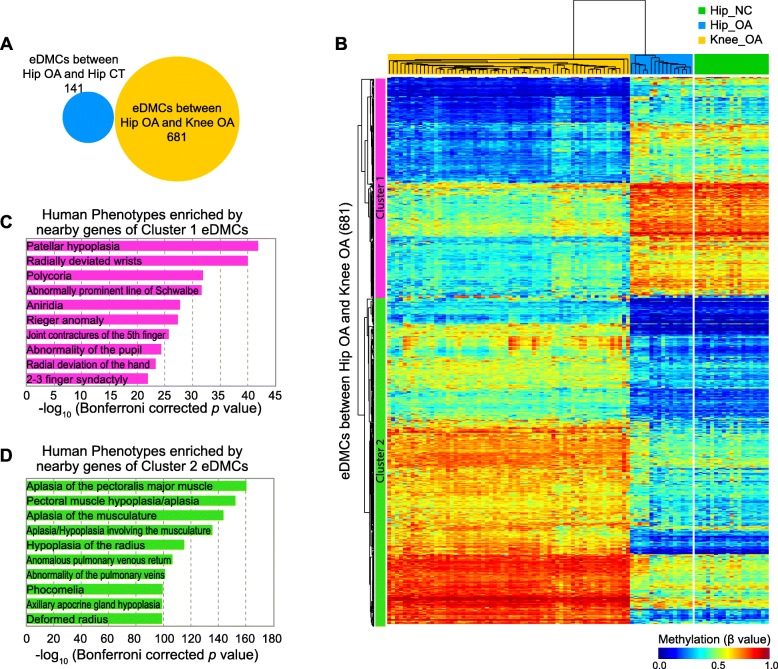
Organ source-dependent differences in enhancer methylation between patients with hip OA and knee OA. **a** The eDMCs between hip OA and knee OA did not overlap with those between hip OA and hip CT. **b** Two-way hierarchical clustering of methylation in the eDMCs between hip OA and knee OA. Each row represents an eDMC, and each column represents a sample. The methylation levels of these eDMCs in the hip CT group are shown according to the row order determined by hierarchical clustering. DNA methylation (β value) is represented by a color from dark blue (unmethylated) to dark red (fully methylated). **c** The top 10 human phenotypes enriched in the genes near the eDMCs in cluster 1 in Figure **b**. **d** The top 10 human phenotypes enriched in the genes near the eDMCs in cluster 2 in Figure **b**

Next, we tried to find the different methylation patterns between the patients with hip OA and knee OA. To this end, we compared the methylation differences in the CpG sites between two groups of patients and identified 681 eDMCs. None of these eDMCs showed methylation differences between the tissues from the patients with hip OA and healthy hip tissues (Fig. 5a). Two-way hierarchical clustering revealed a similar methylation pattern of these eDMCs between tissues from patients with hip OA and healthy hip tissues (Fig. 5b). These results suggested that the differences in enhancer methylation between the hip OA and knee OA groups was organ source-dependent. We noted that all eDMCs were classified into two clusters. The eDMCs in cluster 1 showed lower methylation levels in patients with knee OA, while those in cluster 2 showed lower methylation levels in the hip OA or CT samples. As reported previously, low methylation of enhancers can regulate tissue-specific expression of tissue marker genes. To determine the functions of these eDMCs in two clusters, we carried out functional enrichment based on GREAT software. As shown in Fig. 5c and d, the eDMCs of both clusters were significantly enriched in different human phenotypes (Additional file 6: Table S4). The eDMCs that were poorly methylated in the patients with knee OA were enriched in human phenotypes related to multiple joint abnormalities, while those in the patients with hip OA were enriched in human phenotypes related to muscle hypoplasia and phocomelia. These results suggest the potential roles of eDMCs in activating enhancers of genes in an organ-specific manner.

## Discussion

DNA methylation has important roles in gene regulation in development and human diseases [25]. In particular, abnormal methylation in gene promoter regions induced abnormal expression of disease-related genes [26]. Recent studies have also reported the abnormal methylation of some gene promoters in osteoarthritis. However, the methylation dynamics in enhancers is still unclear. To this end, we systematically analyzed the methylation dynamics in enhancers in two kinds of osteoarthritis, knee osteoarthritis and hip osteoarthritis.

Our analysis of methylation differences between the osteoarthritis and control groups revealed that enhancers undergo substantial DNA methylation changes in both kinds of osteoarthritis. Nearly half (8111) of the differentially methylated CpGs were localized in enhancer regions. This finding highlighted the important roles of enhancers in the development of osteoarthritis for the first time. This comprehensive enhancer methylation atlas is useful for further analysis of the epigenetic regulation of osteoarthritis and the development of clinical drugs for osteoarthritis therapy.

As reported in previous studies, DNA methylation can be used as a stable marker for specific tissues or diseases. Our analysis revealed the major DNA methylation changes related to osteoarthritis in enhancer regions, with a specific focus on the subtyping of osteoarthritis and the influence on human phenotypes. Consistent with these findings, our study identified new subtypes of osteoarthritis based on enhancer methylation profiles, both in knee osteoarthritis and hip osteoarthritis, and revealed that enhancer methylation can be used as a biomarker of osteoarthritis subtypes. For hip osteoarthritis patients, two subtypes were identified, and the main influencing factor was gender. Our study showed that hip osteoarthritis displayed a gender-specific methylation pattern in 2426 differentially methylated CpGs from enhancers, whose nearby genes play important roles in human phenotypes related to osteoarthritis and abnormalities of the hip bone. According to clinical statistics, the osteoarthritis rate in women is much higher than in men, especially after age 55 [3, 27]. To understand the mechanisms underlying this difference, scientists have identified multiple risk factors, including biological factors, genetic predisposition, hormones, and obesity [28]. Our study identified an effect of gender at the molecular level. Enhancers can enhance the expression of target genes by recruiting active chromatin factors in a tissue type-specific manner [9]. Low methylation is needed for the formation of an open chromatin structure in enhancer regions [29]. Thus, the low methylation in females may be the cause of the abnormal activation of enhancers found in osteoarthritis phenotypes. Further analysis of the reasons for gender-specific methylation in hip osteoarthritis would be helpful for further elucidation of the high risk of osteoarthritis in females.

Enhancer methylation dynamics contribute to osteoarthritis plasticity. Thus, targeting DNA methylation may be an alternative therapy for osteoarthritis, similar to targeting DNA methylation for cancer therapy [30]. Currently, the symptoms of osteoarthritis can usually be effectively managed with lifestyle changes, physical therapies, medications, and surgery. However, the process underlying osteoarthritis cannot be reversed. Drugs targeting methylation can change the methylation status in patients with osteoarthritis, which may help reverse osteoarthritis. In particular, editing technology targeting methylation enables modification of the DNA methylation status but has only been studied in infection, which provides a rationale for targeting therapy for osteoarthritis.

## Conclusions

Our analyses indicate that aberrant DNA methylation of enhancers is related to osteoarthritis phenotypes, and our comprehensive enhancer methylation atlas is useful for further analysis of the epigenetic regulation of osteoarthritis and the development of clinical drugs for osteoarthritis therapy.

## Supplementary information

**Additional file 1: Figure S1.** Heatmap showing the similarity in methylation between male patients with hip OA and cancer cell lines. Each row represents a gender-related eDMC between males and females. The order of the rows is the same as that in Fig. 3b. Each column represents a sample, including hip OA samples, healthy hip samples, and multiple cancer cell lines obtained from the ENCODE project at https://hgdownload-test.gi.ucsc.edu/goldenPath/hg19/encodeDCC/wgEncodeHaibMethyl450/supplemental/wgEncodeHaibMethyl450BetaValues.txt. Hierarchical clustering was carried out to show the similarity in methylation between hip OA and the cancer cell lines.

**Additional file 2: Figure S2.** Age distribution of the patients with knee OA in groups 1 and 2 in Fig. 4c. For each group, the patients were classified into male and female groups. Box plots show the distribution of age in each group, and the dots represent the age of each patient.

**Additional file 3: Table S1.** Summary of enhancer CpGs.

**Additional file 4: Table S2.** Function enrichement of eDMCs of cluster 2 in Fig. 2c.

**Additional file 5: Table S3.** Function enrichement of eDMCs between two Knee OA groups.

**Additional file 6: Table S4.** Function enrichement of eDMCs of cluster 1 and 2 in Fig. 4b.

## Data Availability

The DNA methylation data analyzed were downloaded from the GEO database under access ID GSE63695.

## Abbreviations

eDMCs: enhancer differentially methylated CpGs
OA: Osteoarthritis
TSSs: Transcript start sites
