# Supplementary material for: Genome-wide analysis of aberrant methylation of enhancer DNA in human osteoarthritis
Source: BMC Med Genomics. 2020 Jan 3;13:1. doi: 10.1186/s12920-019-0646-9 (PMC6942377; doi:10.1186/s12920-019-0646-9)
Supplement: Supplementary file 1 — Additional file 1: Figure S1. Heatmap showing the similarity in methylation between male patients with hip OA and cancer cell lines. Each row represents a gender-related eDMC between males and females. The order of the rows is the same as that in Fig. 3b. Each column represents a sample, including hip OA samples, healthy hip samples, and multiple cancer cell lines obtained from the ENCODE project at https://hgdownload-test.gi.ucsc.edu/goldenPath/hg19/encodeDCC/wgEncodeHaibMethyl450/supplemental/wgEncodeHaibMethyl450BetaValues.txt. Hierarchical clustering was carried out to show the similarity in methylation between hip OA and the cancer cell lines. [file 12920_2019_646_MOESM1_ESM.pdf]

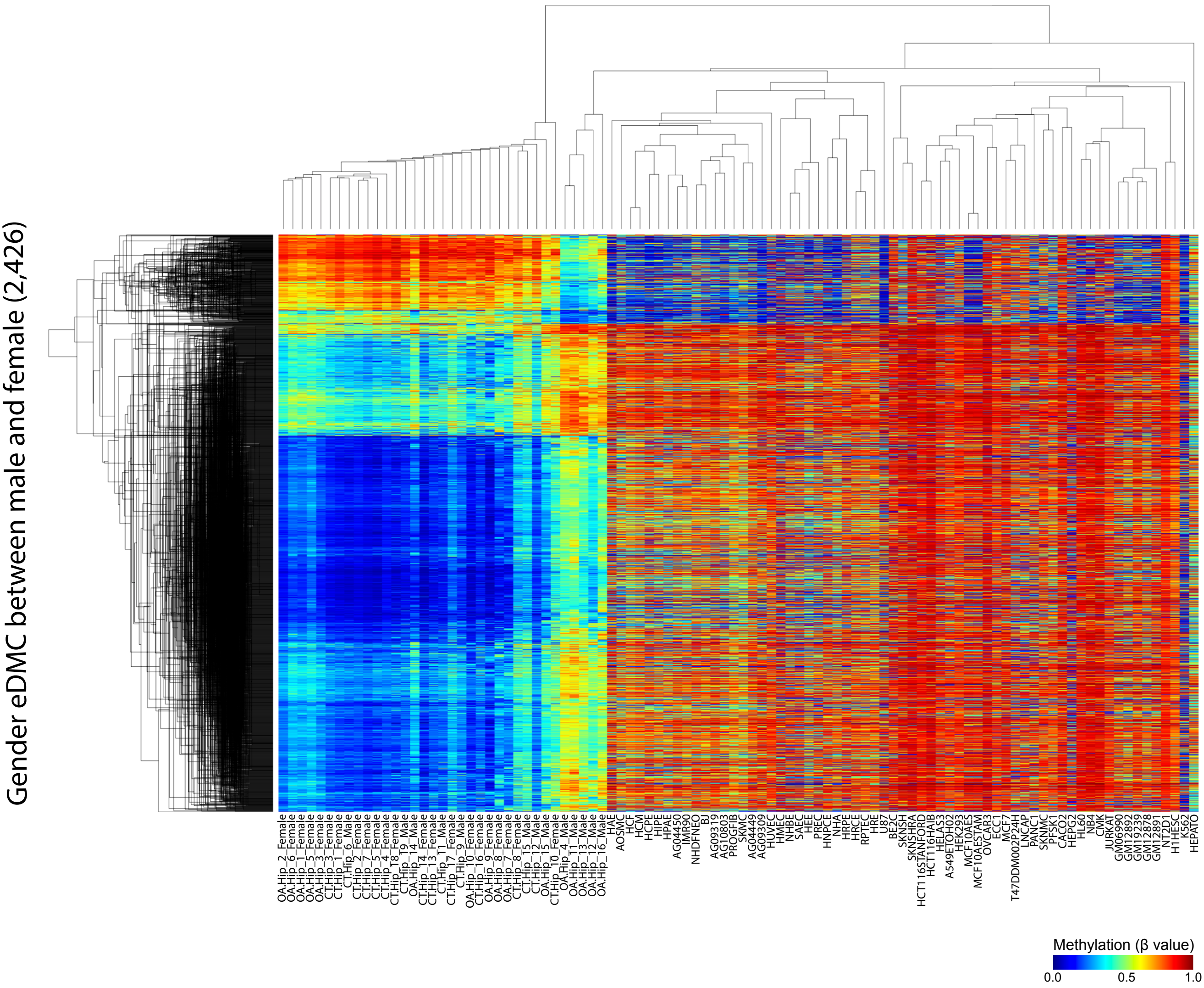

Supplemental Figure 1. Heatmap showed the methylation similarity betwen male Hip OA patients and cancer cell lines. Each row represents a gender eDMC between male and female. The order of rows is the same with Figure 2B. Each column represents a samples including Hip OA, normal Hip, and mutiple cancer cell lines obtained from ENCODE project at https://hgdownload-test.gi.ucsc.edu/goldenPath/hg19/encodeDC-C/wgEncodeHaibMethyl450/supplemental/wgEncodeHaibMethyl450BetaValues.txt. Hierarchical clustering was carried out to show the methylation similarity between Hip OA and cancer cell lines.
