# Supplementary material for: Genome-wide analysis of aberrant methylation of enhancer DNA in human osteoarthritis
Source: BMC Med Genomics. 2020 Jan 3;13:1. doi: 10.1186/s12920-019-0646-9 (PMC6942377; doi:10.1186/s12920-019-0646-9)
Supplement: Supplementary file 2 — Additional file 2: Figure S2. Age distribution of the patients with knee OA in groups 1 and 2 in Fig. 4c. For each group, the patients were classified into male and female groups. Box plots show the distribution of age in each group, and the dots represent the age of each patient. [file 12920_2019_646_MOESM2_ESM.pdf]

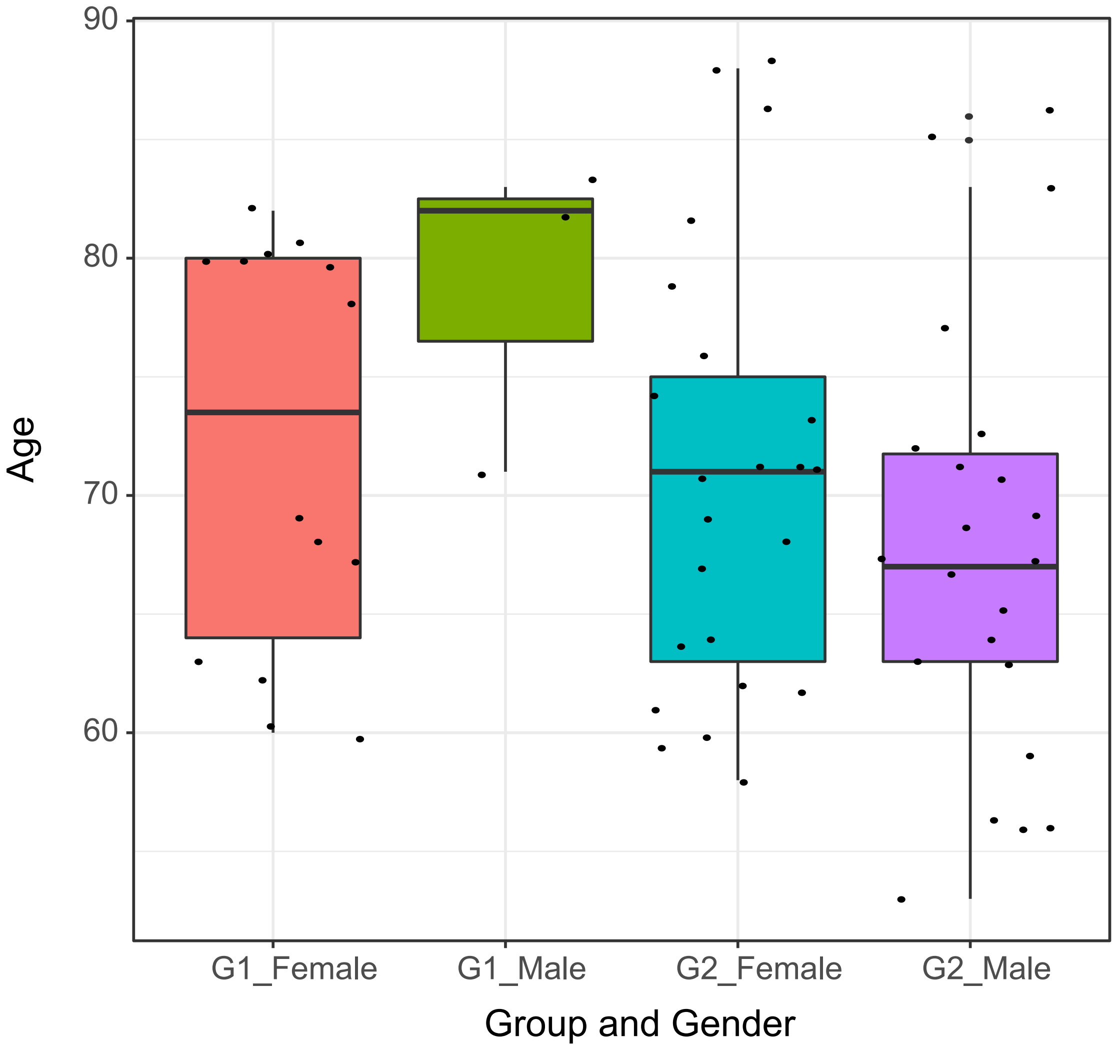

Supplemental Figure 2. Age distribution of Knee OA patients in group 1 and 2 in Figure 3C. For each group, the patients were classified into male and female groups. Box plots show the distribution of age in each group and the dots represent the age of each patient.
